# Therapeutic hypothermia modulates the neurogenic response of the newborn piglet subventricular zone after hypoxia-ischemia

**DOI:** 10.1038/s41390-023-02751-7

**Published:** 2023-08-12

**Authors:** Daniel Alonso-Alconada, Pierre Gressens, Xavier Golay, Nicola J. Robertson

**Affiliations:** 1 Department of Cell Biology & Histology, School of Medicine & Nursing, University of the Basque Country (UPV/EHU), Barrio Sarriena s/n, 48940 Leioa, Bizkaia Spain; 2 Université Paris Cité, NeuroDiderot, Inserm, F-75019 Paris France; 3 Department of Brain Repair and Rehabilitation, Institute of Neurology, University College London, London, WC1N 3BG UK; 4 Institute for Women’s Health, University College London, London, UK; 5 Edinburgh Neuroscience & Centre for Clinical Brain Sciences (CCBS), The University of Edinburgh, Chancellor’s Building, 49 Little France Crescent, Edinburgh, EH16 4SB* UK

## Abstract

**Background:**

Neuroprotection combined with neuroregeneration may be critical for optimizing functional recovery in neonatal encephalopathy. To investigate the neurogenic response to hypoxia-ischemia (HI) followed by normothermia (38.5 °C) or three different hypothermic temperatures (35, 33.5, or 30 °C) in the subventricular zone (SVZ) of the neonatal piglet.

**Methods:**

Following transient cerebral HI and resuscitation, 28 newborn piglets were randomized to: normothermia or whole-body cooling to 35 °C, 33.5 °C, or 30 °C during 2–26 h (all *n* = 7). At 48 h, piglets were euthanized and SVZ obtained to evaluate its cellularity, pattern of cell death, radial glia length, doublecortin (DCX, neuroblasts) expression, and Ki67 (cell proliferation) and Ki67/Sox2 (neural stem/progenitor dividing) cell counts.

**Results:**

Normothermic piglets showed lower total (Ki67+) and neural stem/progenitor dividing (Ki67+Sox2+) cell counts when compared to hypothermic groups. Cooling to 33.5 °C obtained the highest values of SVZ cellularity, radial glia length processes, neuroblast chains area and DCX immunohistochemistry. Cooling to 30 °C, however, revealed decreased cellularity in the lateral SVZ and shorter radial glia processes when compared with 33.5 °C.

**Conclusions:**

In a neonatal piglet model, hypothermia to 33.5 °C modulates the neurogenic response of the SVZ after HI, highlighting the potential beneficial effect of hypothermia to 33.5 °C on endogenous neurogenesis and the detrimental effect of overcooling beyond this threshold.

**Impact:**

- Neuroprotection combined with neuroregeneration may be critical for optimizing functional recovery in neonatal encephalopathy.
- Hypothermia may modulate neurogenesis in the subventricular zone (SVZ) of the neonatal hypoxic-ischemic piglet.
- Cooling to 33.5 °C obtained the highest values of SVZ cellularity, radial glia length processes, neuroblast chains area and doublecortin immunohistochemistry; cooling to 30 °C, however, revealed decreased cellularity and shorter radial glia processes.
- In a neonatal piglet model, therapeutic hypothermia (33.5 °C) modulates the neurogenic response of the SVZ after hypoxia-ischemia, highlighting also the detrimental effect of overcooling beyond this threshold.

## Introduction

Neonatal encephalopathy is an important cause of brain injury in term and near-term infants, affecting over 1 million babies worldwide.^1^ Therapeutic hypothermia (HT) is currently the only neuroprotective therapy that improves neurodevelopmental outcomes in large trials involving humans;^2,3^ reducing the core temperature of at-risk infants to 33.5 °C for 72 h, is standard care for moderate-severe neonatal encephalopathy following suspected hypoxia-ischemia (HI) in high resource settings.

Mechanisms through which HT is neuroprotective following HI include a reduction in brain metabolic rate, a drop in oxygen consumption rate, immunomodulation and an amelioration of free radical production and subsequent reduction in cell death.^4,5^ As current protocols seem optimal for neuroprotection,^6,7^ the possible modulation of the neurogenic response through HT is of great interest.

Postnatally, two neurogenic areas persist in the brain where new neurons are produced from stem or progenitor cells: the subgranular zone of the dentate gyrus of the hippocampus and the subventricular zone (SVZ) of the lateral ventricles.^8,9^ Cells from the SVZ have been molecularly manipulated in situ to induce their proliferation and migration to the sites of damage or in vitro to subsequently be transplanted.^8,10–12^

Based on this, in vivo modulation of the neurogenic potential of the SVZ may contribute to brain tissue remodeling after damage. However, the relationship described between hypothermia and neurogenesis in the neonatal brain is controversial. In neonatal rats, Xiong et al. described a neuroregenerative role of HT after HI, as animals maintained at 32–33 °C for 24 h showed higher newborn immature and mature neurons compared with normothermia group.^13^ However, using a shorter cooling protocol (32 °C for 5 h), Matsuda et al. were not able to observe a modulation of the neuroblast marker doublecortin.^14^ Further, a severe hypothermic environment (30 °C for 24 h) induced a decrease of neurogenesis in the neonatal rat,^15^ suggesting the potential risk of adverse effects of severe hypothermia on the nervous system. In fetal sheep, HI-induced decrease in cell proliferation was not restored despite hypothermic treatment;^16^ however, authors did not evaluate the neurogenic niches but periventricular white matter, so the effect of HT on the neurogenic response after HI in large animal models remains far from clear.

The aim of this study was to investigate the neurogenic response of the piglet SVZ after neonatal HI followed by i) normothermia (38.5 °C) or ii) a 24 h duration of HT at three different hypothermic target temperatures (35 °C, 33.5 °C or 30 °C).

## Material and methods

The experimental protocol was approved by the Animal Care and Use Committee of University College London Biological Services and Institute of Neurology and conducted according to UK Home Office Guidelines (Animals [Scientific Procedures] Act 1986).

### Animal experiments and surgical preparation

Twenty-eight large white male piglets aged <24 h were subjected to a controlled hypoxic-ischemic insult. Animal preparation and surgical experiments have been described previously in deep.^7,17^ In brief, piglets were sedated with intramuscular midazolam (0.2 mg/kg), and we monitored arterial O_2_ saturation (Nonin Medical). With the help of a facemask, we applied isoflurane anesthesia (4% vol/vol) during tracheostomy and intubation, which was later maintained (3% during surgery, 2% otherwise). Animals were mechanically ventilated so as to maintain the arterial pressures of O_2_ (PaO_2_; 8–13 kPa) and CO_2_ (PaCO_2_; 4.5–6.5 kPa) allowing for temperature correction of the arterial blood sample. To infuse maintenance fluids (10% dextrose, 60 ml/kg/d), fentanyl (3–6 μg/kg/h), and antibiotics (benzylpenicillin 50 mg/kg and gentamicin 2.5 mg/kg, every 12 h), an umbilical venous catheter was inserted.

With an umbilical arterial catheter, we continuously monitorized heart rate (HR) and mean arterial blood pressure (MABP) and obtained 6-h blood sampling to measure PaO2, PaCO2, pH, electrolytes, glucose (3–10 mmol/l), and lactate (Abbott Laboratories). Bolus infusions of colloid (Gelofusin, B Braun Medical Ltd.) and inotropes maintained MABP > 40 mmHg. Arterial lines were maintained by infusing 0.9% saline solution (Baxter, 1 ml/h) with heparin sodium (1 IU/ml) to prevent line blockage. To later induce ischemia, both common carotid arteries were surgically isolated at the level of the fourth cervical vertebra and encircled by remotely controlled vascular occluders (OC2A, In Vivo Metric). After surgery, animals were positioned prone in a plastic pod with their heads immobilised.

### Cerebral HI

Transient HI was induced by (i) inflating the vascular occluders, thus inducing the remote occlusion of both common carotid arteries, and (ii) reducing the fraction of inspired oxygen (FiO_2_) to 12% (vol/vol).

During HI, cerebral energetics were monitored every 2 min by phosphorus (^31^P) magnetic resonance spectroscopy (MRS), and the β-nucleotide triphosphate (β-NTP; mainly ATP) peak height was automatically measured. When β-NTP peak height had fallen to 40% of baseline, FiO_2_ was adjusted to stabilize β-NTP at that level for 12.5 min. At the end of this 12.5-min period, the occluders were deflated and FiO_2_ was normalized. ^31^P spectra were acquired for a further 1 h to monitor recovery from HI. The time integral of the decrement of β-NTP/EPP (EPP = exchangeable phosphate pool = inorganic phosphate + phosphocreatine + (2γ + β)-NTP) during HI and the first 1 h of resuscitation quantified the acute energy depletion.

### Experimental groups

After HI and resuscitation, and using a computer-generated randomization sequence and opaque sequentially numbered envelopes, piglets were randomized into 4 groups (all *n* = 7): (i) normothermia (rectal temperature [Trec], 38.5 °C throughout) or whole-body cooling from 2 to 26 h after insult to Trec (ii) 35 °C, (iii) 33.5 °C, or (iv) 30 °C. Normothermic piglets were maintained at their target Trec using a warmed water mattress (Tecotherm) above and below the animal. Hypothermic piglets were cooled (by reducing the water mattress temperature) to their target Trec over 90 min starting 2 h after HI. At 26 h after HI, cooled piglets were rewarmed to normothermia at 0.5 °C/h using a water mattress with circulating water heated to increasing temperatures. All animals received continuous physiological monitoring (SA instruments) and intensive life support throughout experimentation.

### Brain sampling

Piglets were euthanized at 48 h after HI with intravenous pentobarbital and the brain fixed by cardiac perfusion with cold 4% paraformaldehyde, dissected out and post-fixed at 4 °C in 2% paraformaldehyde. Two coronal slices (5 mm thick) from the right hemisphere at the level of the optic chiasm were embedded in paraffin and sectioned to 5-µm thickness.

**Fig. 1:**
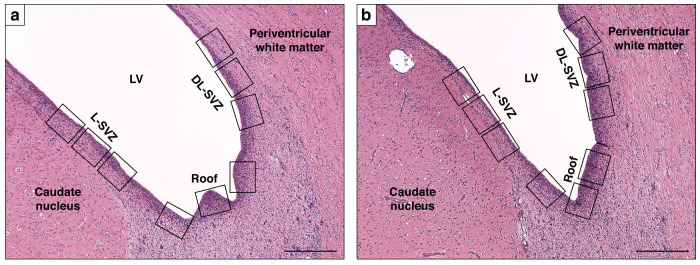
Low magnification images showing the SVZ the neonatal piglet and its 3 subareas: the roof, the lateral-SVZ (L-SVZ, apposed to the caudate nucleus) and the dorsolateral-SVZ (DL-SVZ, close to the periventricular white matter). Representative microphotographs of coronal section of the piglet SVZ at bregma −2.0 (**a**) and −4.0 (**b**) brain levels sampled in this study. H&E staining. LV lateral ventricle, SVZ subventricular zone, DL-SVZ dorsolateral-SVZ, L-SVZ lateral-SVZ. Original magnification 40x. Scale bar: 500 µm.

For each animal, 3 non-consecutive sections from 2 levels (bregma −2.0 and −4.0) were evaluated. The region of interest was the SVZ, which was in turn divided into 3 subareas (Fig. 1): the roof, the lateral-SVZ (L-SVZ, apposed to the caudate nucleus) and the dorsolateral-SVZ (DL-SVZ, close to the periventricular white matter).

### Histological evaluation of cellularity and of pattern of cell death

Paraffin-embedded brain samples were stained with Hematoxilin-Eosin (H&E; Shandon Varistain® V24-4, Thermo Electron Corporation) to study cellularity and the pattern of cell death in the roof, L-SVZ and DL-SVZ subareas of the SVZ by using Fiji/Image J image software. For each animal, level and section, a total of nine non-overlapping microphotographs (3 from each subarea of the SVZ, Fig. 1) were taken at X400 magnification in a light field optical microscope (Olympus BX50F4, Japan). Necrotic cells were characterized by a pyknotic nucleus or no nucleus, along with a swollen, eosinophilic cytoplasm. Apoptotic-like cells, in turn, were identified by the presence of nuclear karyorrhexis and low cytoplasmic change.^18^ To exclude apoptotic/necrotic endothelial cells or white blood cells, we did not count these cell phenotypes when close to or within blood vessels. Together with cells with necrotic or apoptotic features, we also counted morphologically well-preserved/undamaged cells, and the results were averaged from 3 high-power fields from 3 slides from the same region in each animal. All these analyses were performed by a histologist blinded to the samples and values given as cells per mm^2^.

### Immunohistochemistry

Paraffin embedded slices were deparaffined and antigen retrieval carried out by incubating the samples in a 10 mM sodium citrate solution at pH 6 + 0.05% Tween20 in distilled water, where they were boiled 3 times, maintained at 95–98 °C for 20 min and finally cooled at room temperature. After blocking in 5% bovine serum albumin buffer, brain samples were incubated overnight with the following primary antibodies: mouse anti-GFAP (1:100, MA5-12023, Thermo Fisher) to label radial-glia/neural stem cells and then assess neurogenic activity close to the ventricular wall; mouse anti-DCX (doublecortin,1:50, sc-271390, Santa Cruz Biotechnology) to identify young neurons/neuroblasts; and mouse anti-Ki67 (1:50, STJ96966, St Johns Labs, UK) to label dividing cells/proliferation. To study the proliferation of neural stem/progenitor cells, we performed a double immunohistochemistry with Ki67 and Sox2 (1:100, Santa Cruz Biotechnology) for cell colocalization. The day after, brain samples were incubated with Alexa Fluor 488 and/or Texas Red secondary antibodies (both 1:300, Thermo Fisher) for 1 h at room temperature in the dark, and, after several washes, slices were mounted (F4680, Sigma). Negative controls received identical treatment except for omission of primary antibodies and showed no specific staining.

### Immunohistochemical evaluation

Sections were examined by an investigator blind to the treatment group and analyzed with Fiji/Image J image software.

For GFAP+ cells processes, Ki67+ and Ki67+Sox2+ evaluation, nine non-overlapping microphotographs (3 from each SVZ sub-area, rectangles in Fig. 1) were taken with a fluorescence laser microscope at X200 or X400 magnification from each animal, level and section. The length of the GFAP+ cells processes lining the SVZ was measured and averaged at 400X magnification after 6 measurements for each photograph including the longest 6 processes of the region.^19^ Values are given as µm.

GFAP labeling area % was quantified using Fiji/Image J software following the protocol developed by ref. ^20^ Pixels with intensity values approximately like the background were replaced with the mean background intensity value using the subtract background tool equal to 50. A label mask was created using the adjust threshold tool and the Otsu algorithm. Finally, the area of the immunolabelling was divided by the total area of the region of interest imaged to obtain the area %.

Ki67 positive and Ki67/Sox2 double positive cells were counted in 3 non-adjacent fields of view (rectangles in Fig. 1) at x200 magnification along the roof, L-SVZ and DL-SVZ edges of the lateral ventricle.^19,21^ In each case, the total number of Ki67-positive and Sox2/Ki67 double positive cells in the roof, L-SVZ and DL-SVZ sub-regions were counted and divided by the area to obtain values of cells per mm^2^.

For DCX evaluation, all the immunopositive areas along the SVZ were digitalized and whole fluorescence analyzed because of the confluence of the expression.^22^ Using H&E-stained sections, we also measured the area of neuroblast chains in the SVZ (values are given as mm^2^). This high density of cells that take up hematoxylin correspond to DCX.^23^

### Statistical analysis

All results are given as mean ± 95% CI. Parametric data were analysed using a one-way analysis of variance (ANOVA) with Tukey post hoc test, whereas Kruskal–Wallis test was used for non-parametric data. Pearson correlation analysis and calculation of a coefficient of determination (R^2^) were used to determine the relationship between the area of neuroblast chains evaluated with H&E staining and DCX immunofluorescence. Analysis was performed using GraphPad PRISM8 (GraphPad Software, Inc., La Jolla, CA). A value of *p* < 0.05 was taken as statistically significant.

## Results

No piglet died in the normothermic, 35 or 33.5 °C-cooled groups. One animal cooled to 33.5 °C suffered two cardiac arrests but survived to 48 h. Five piglets cooled to 30 °C died before 48-h post-HI and were not included in the study.

### SVZ’s cellularity was higher with HT at 33.5 °C, with no differences in cell death between groups

**Fig. 2:**
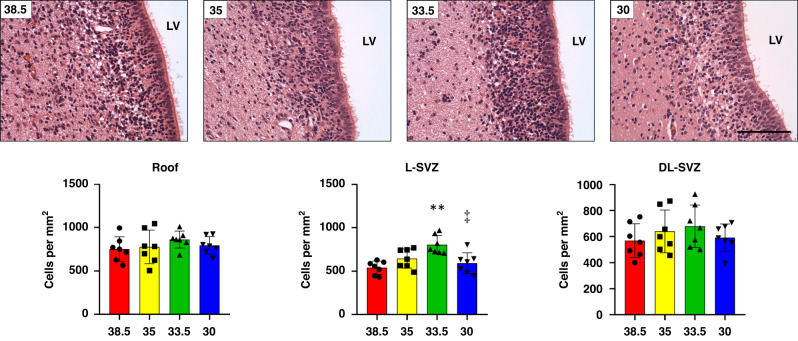
Representative microphotographs of the subventricular zone (SVZ) from HI and normothermia (38.5 °C) or HT-treated piglets at 35, 33.5 or 30 °C. In the L-SVZ, HT at 33.5 °C showed higher number of cells than normothermic (**p < 0.01) or HT at 30 °C (^‡^*p* < 0.01) animals, whereas the roof and the DL-DVZ showed no significant differences. No differences in cell counts were found between normothermia (38.5 °C) or HT at 35 or 30 °C. LV lateral ventricle, SVZ Subventricular zone, L-SVZ lateral-SVZ, DL-SVZ dorsolateral-SVZ. H&E staining. Original magnification 400x. Scale bar: 100 µm.

We first evaluated the cellularity of the piglet SVZ after HI and normothermia (38.5 °C) or hypothermia at 35 °C, 33.5 °C or 30 °C. The SVZ consists of a high-density population of small darkly stained cells located immediately adjacent to the ventricles. Using H&E staining (Fig. 2), we quantified the number of morphologically well-preserved cells in the roof and along the length of the lateral (L-SVZ, close to the caudate nucleus) and dorsolateral (DL, close to the periventricular white matter) walls of the lateral ventricle. In the L-SVZ, cooling at 33.5 °C showed higher number of cells than normothermic (38.5 °C, **p < 0.01) and cooled at 30 °C (^‡^*p* < 0.01) animals (Fig. 2, graph), whereas the roof and the DL-SVZ revealed no significant differences in cell counts. No differences in cell counts were found between normothermia (38.5 °C) or hypothermia at 35 or 30 °C.

Cells with necrotic or apoptotic features were also studied in the four groups, showing no differences in cell counts for any (data not shown), neither in the three SVZ subareas evaluated (roof, DL-SVZ, or L-SVZ): there was minimal or absent cell death in the SVZ, with low values for necrotic- (0–0.7 cells per mm^2^) or apoptotic-like cells (0.6–1.3 cells per mm^2^) after HI and normothermia or hypothermia at any target temperature.

### HT to 33.5 °C obtained the highest values of radial glia/GFAP+ cells length processes

**Fig. 3:**
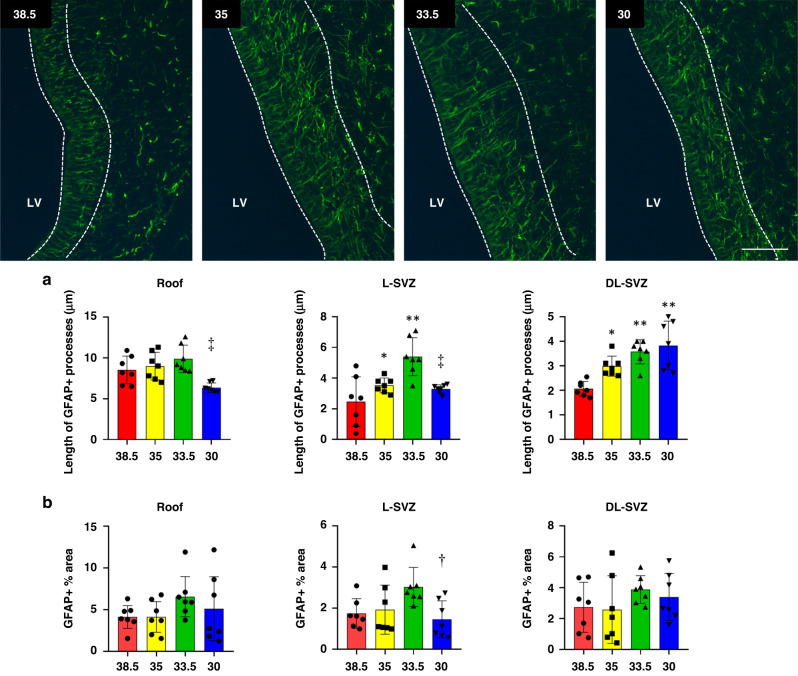
Fluorescent microphotographs of GFAP immune-stained samples from the piglet SVZ after HI and normothermia (38.5 °C) or HT at 35, 33.5 or 30 °C. **a** GFAP reveals the length of the processes of radial-glia/neural stem cells in the SVZ. The length of GFAP+ processes after HI and normothermia (38.5 °C) was shorter when compared with HT at 33.5 °C (**p < 0.01 in the L-SVZ and DL-SVZ) and at 35 °C (*p < 0.05 in the DL-SVZ and L-SVZ). HT at 30 °C obtained lower values (^‡^*p* < 0.01) than 33.5 °C in both the roof and the L-SVZ. **b** Quantification of GFAP expression using densitometry. The highest values of GFAP coverage (expressed as GFAP % area in the graphs) were observed in 33.5 °C animals for the three areas evaluated; however, there were no significant differences in GFAP coverage between normothermia (38.5 °C) and cooling to any temperature. HT at 30 °C obtained significant lower values (^†^*p* < 0.05) than 33.5 °C in the L-SVZ. LV, lateral ventricle. Original magnification 400x. Scale bar: 100 µm.

To assess the neurogenic activity close to the ventricular wall, we immunostained the SVZ with GFAP as a surrogate for radial glia (Fig. 3). These GFAP+ cells (radial-glia like cells or type 1 or neural stem cells) showed a triangular shape and a large cytoplasm with thin processes, which we measured in the three SVZ subareas (i.e., roof, L-SVZ and DL-SVZ) and the four temperature conditions.

The length of these GFAP+ processes was longer in the roof and decreased in size when distancing from this area along the L-SVZ and DL-SVZ. In the roof and L-SVZ, the highest values were obtained for the 33.5 °C group (Fig. 3a). In the roof, there were no differences between the 38.5, 35, or 33.5 °C groups, whereas the 30 °C group showed shorter processes when compared to 33.5 °C (^‡^*p* < 0.01). In the L-SVZ, the measurements of the 33.5 °C group were double those of the normothermic (38.5 °C) piglets (**p < 0.01). As in the roof, the 30 °C group showed shorter processes in the L-SVZ when compared to 33.5 °C (^‡^*p* < 0.01), with no statistical differences with normothermic animals. In the DL-SVZ, the lowest values were reported in normothermic animals, whose processes were statistically shorter than those observed for the 35 (*p < 0.05), and 33.5 and 30 °C (**p < 0.01) groups.

We also evaluated GFAP coverage (expressed as GFAP % area, in the graphs) using densitometry (Fig. 3b). Despite HT at 33.5 °C showed the highest values of GFAP coverage in the three areas evaluated, there were not significant differences between normothermic (38.5 °C) and cooled animals at any temperature. HT at 30 °C obtained significant lower values (^†^*p* < 0.05) than 33.5 °C in the L-SVZ.

### Normothermic animals showed lower counts of total and neural stem/progenitor dividing cells in the SVZ

**Fig. 4:**
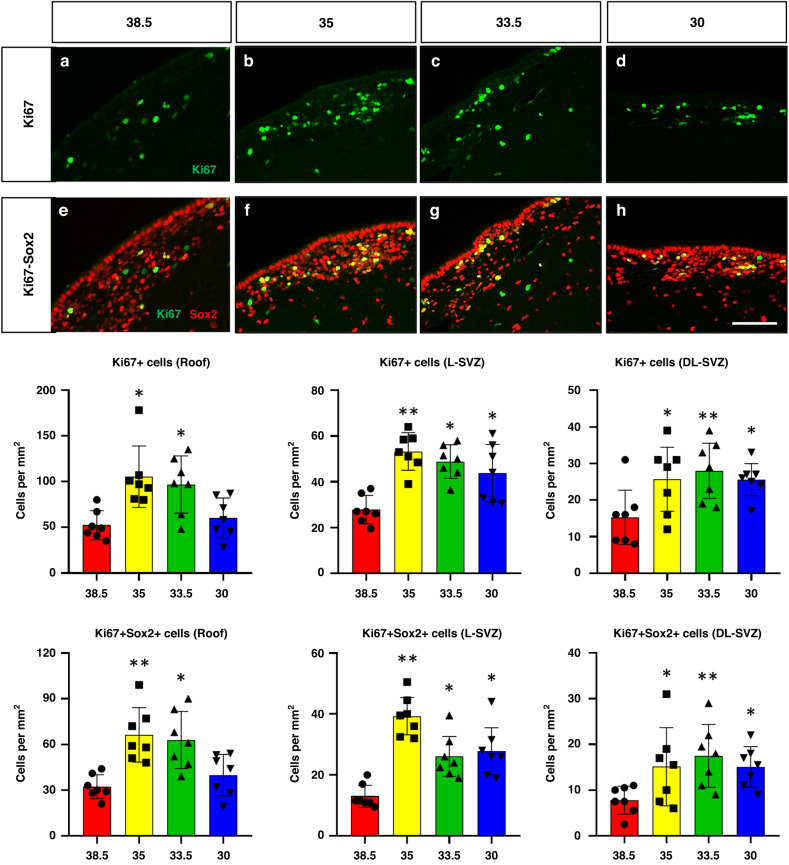
Fluorescent microphotographs of Ki67 and Ki67/Sox2 immune-stained samples from the piglet SVZ after HI and normothermia (38.5 °C) or HT at 35, 33.5 or 30 °C. Cell proliferation (Ki67+, green, **a**–**d**) and neural stem/progenitor cell proliferation (Ki67+Sox2+, yellow, **e**–**h**) evaluation in the roof, L-SVZ and DL-SVZ of the neonatal piglet 48 h after HI and normothermia (38.5 °C, **a**, **e**) or HT at 35 (**b**, **f**), 33.5 (**c**, **g**) or 30 °C (**d**, **h**). HI and normothermia (38.5 °C) showed low values of Ki67+ and Ki67+Sox2+ cells in the roof, L-SVZ and DL-SVZ. HT to any temperature (*p < 0.05 vs 38.5 or **p < 0.01 vs 38.5) attenuated the inhibition of dividing cells and neural stem/progenitor cell proliferation in the roof, L-SVZ and DL-SVZ of the neonatal piglet after HI. Original magnification 400x. Scale bar: 100 µm.

The proliferative capacity of the SVZ after HI was evaluated by Ki67 (Fig. 4a–d), revealing that the normothermic group (38.5 °C) displayed fewer dividing cells (*p < 0.05 or **p < 0.01) in the L-SVZ and DL-SVZ than any of the three hypothermic target temperatures. In the roof, hypothermia to 35 or to 33.5 °C obtained higher Ki67+ cell counts when compared to normothermic animals (*p < 0.05), whereas cooling at 30 °C did not show increased cell proliferation.

Neural stem/progenitor cell proliferation, determined by Ki67 and Sox2 double labeling (Fig. 4e–h), was also affected after HI and normothermia, with significant (*p < 0.05 or **p < 0.01) lower cell counts in the normothermic L-SVZ and DL-SVZ if compared to any of the three hypothermia groups. In the roof, hypothermia to 35 (**p < 0.01) or to 33.5 °C (*p < 0.05) obtained higher cell counts for Ki67+Sox2+ when compared to normothermic animals, whereas cooling at 30 °C did not show increased cell counts. There were no statistical differences in the number of Ki67+ or Ki67+Sox2+ cells between cooled groups.

### Cooling to 33.5 °C obtained the highest values when analysing neuroblasts

**Fig. 5:**
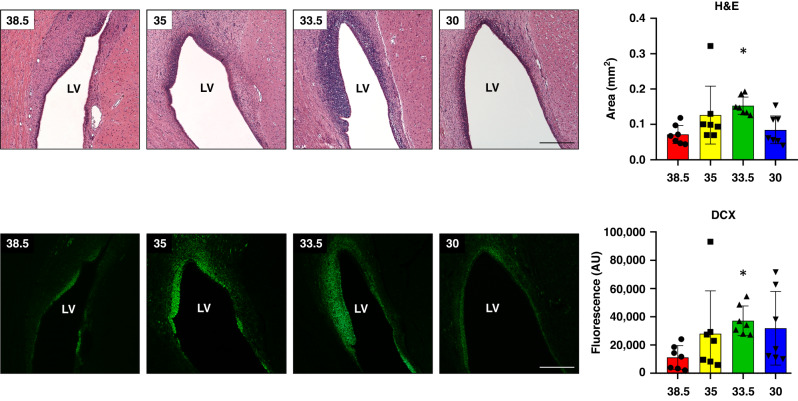
Neuroblast evaluation through H&E staining and DCX immunohistochemistry in the SVZ of the neonatal piglet 48 h after HI and normothermia (38.5 °C) or HT at 35, 33.5 or 30 °C. When compared to normothermia (38.5 °C), the only cooling temperature that showed higher values of average neuroblast chains area and fluorescent intensity of DCX expression in the SVZ was HT at 33.5 °C (*p < 0.05 vs 38.5 °C for both H&E and DCX). Original magnification x100. Scale bar: 500 μm.

Normothermic animals demonstrated the lowest values in both neuroblast chain area by H&E and doublecortin (DCX) immunofluorescence (Fig. 5). Only cooling to 33.5 °C showed significantly higher values for both parameters: H&E neuroblast chain area (*p < 0.05 vs normothermia-38.5 °C) and DCX immunostaining (*p < 0.05 vs normothermia-38.5 °C). There was a strong positive correlation between the area of neuroblast chains evaluated by H&E and DCX immunofluorescence (R^2^ = 0.6398, *p* < 0.0001; Supplementary Fig. 1).

## Discussion

Neonatal piglets submitted to HI and maintained normothermic (38.5 °C) or cooled to 35, 33.5, or 30 °C for 48 h showed a different response in the SVZ neurogenic niche. Following HT, the SVZ showed higher cell counts for the proliferative marker Ki67 and for neural stem/progenitor cell proliferation (Ki67/Sox2 double positive cells) when compared to normothermic animals. Further, cooling at 33.5 °C obtained the highest values in SVZ’s cellularity, radial glia length processes, and neuroblasts, thus suggesting that moderate HT modulates the neurogenic response of the piglet SVZ.

The neurogenic response following perinatal HI is unclear. Preclinical studies using rodents have shown an increase in the regenerative capacity of the SVZ after the insult,^24,25^ whereas other studies reported a decrease in cell proliferation.^26,27^ These contradicting results may reflect differences in the severity of the insult, but also in the experimental models used, the temporal profile of the works, or the cellular/molecular methods employed for the assessment of cell proliferation and neurogenesis. In a recent work by our group using the piglet model of neonatal HI,^17^ animals maintained normothermic for 48 h showed a decrease in cellularity and in the number of Ki67+ cells, Ki67+Sox2+ cells and neuroblasts of the SVZ, thus suggesting that, early after the hypoxic-ischemic event, a reduction in SVZ cell proliferation and neurogenesis occurs.

HT is able to modulate a wide range of cellular and molecular processes after acute neurological insults, including the reduction of excitotoxicity and apoptosis and the increase of neurotrophic factors and cell survival pathways and proliferation.^4,5^ Neuroprotection combined with neurorestoration may be critical for optimizing functional recovery in NE. However, information regarding the influence of HT on neurogenesis in the developing brain is limited, despite being the only effective clinical intervention for NE. In a preclinical study in neonatal rats, Xiong et al.^13^ reported that HT to 33 °C for 24 h produced an increase in the number of immature and mature neurons. This effect was accompanied by the blockage of apoptotic cell death in dividing neural stem cells and with the improvement of the local microenvironment, which positively influences the regulation and maintenance of stem cells.^13,28^ In contrast, Lasarzik et al.^29^ suggested that HT might have no effect on neurogenesis after ischemia in adult rats, but their protocol seems to be quite short (45 min), which would have influenced the effect of HT.^30^

Here, hypothermic piglets showed higher counts of total (Ki67+) and neural stem/progenitor dividing (Ki67+Sox2+) cells in the SVZ when comparing to the normothermia group. This observation suggests that HT maintains (or even augments) the cell proliferation rate and the production of neurons from the neurogenic niches, and that this might contribute to functional recovery after HI.^24^ Further, moderate cooling obtained the highest values of SVZ cellularity and radial glia length processes, and the study of neuroblasts confirmed these observations: HT to 33.5 °C obtained the best results. Similarly, moderate hypothermia (32–33 °C) promoted the proliferation of immature neurons detected by DCX in an asphyxia model of cardiac arrest;^30^ it seems that small temperature decreases may facilitate the differentiation of precursor cells.^31^

Optimal neuronal and white matter protection after HI in newborn piglets was seen with moderate cooling (35–33.5 °C), together with substantial brain injury when treated with 30 °C.^7^ Our piglet model has strong similarities to newborn infants with neonatal encephalopathy, in terms of the timing of the evolution of injury after HI,^32,33^ pattern of injury, neuropathology and cerebral magnetic resonance spectroscopy.^34^ Also, a clinical trial evaluating potential benefit of longer cooling, deeper cooling, or both, revealed that deeper cooling was associated with higher use of inhaled nitric oxide therapy, extracorporeal membrane oxygenation, more days of oxygen, and higher incidence of bradycardia. The trial was closed to patient enrollment because of safety and futility concerns.^6^ These data suggest that an effective temperature range exists below which hypothermic neuroprotection (and by extension, neurorestoration) can be lost. In this work, HT to 30 °C reduced the cellularity of the L-SVZ and the length of radial glia processes in the roof and L-SVZ when comparing with 33.5 °C. In line with this, Kanagawa et al.^15^ described a reduction in the cell-proliferation marker bromodeoxyuridine in the neonatal rat when using a hypothermic protocol in which the target temperature was 30 °C. Neuroendocrinological mechanisms are regulated by body temperature. A hypothermic environment produces an increase in corticosteroid levels in plasma,^35^ which can in turn decrease neurogenesis.^36^ In addition, several growth factors have a relevant role in the proliferation and differentiation of neural stem cells, and their expression can be influenced by hypothermia.^15^ It seems that severe instead of moderate hypothermia may suppress cell proliferation and neurogenesis in the developing brain,^15,31^ thus emphasizing on the importance of the hypothermia protocol used.

This study has some limitations. We assessed only the short-term effects of HT on neurogenesis due to the duration of our protocol: the long-term effects are unknown, as well as the fate of the newly formed neurons or their possible integration into the brain circuitry. However, moderate cooling (33 °C) improved neurogenesis 72 h after traumatic brain injury,^37^ with a later increase in DCX levels at 7 days. This continuing improvement was also described in immature rats,^13^ reporting that moderate HT (33 °C) promoted the generation of neural cells 1-week post-HI. The sample size is also a study limitation. Given the small sample size, only male piglets were used to reduce intergroup variation. We were, therefore, not able to examine the increasingly recognized impact of sex on HI outcome.^38^ Inclusion of both sexes is a necessary area for future development of the model, to unravel the potential effect of sex on brain injury, HT and neurogenesis after HI, and will require larger group sizes.

In conclusion, in a neonatal piglet model, HT to 33.5 °C modulates the neurogenic response of the SVZ after HI, highlighting the potential beneficial effect on endogenous neurogenesis of HT to 33.5 °C and the detrimental effect of excessive cooling. These results imply that HT might have potential to ameliorate regional injury by the replacement of damaged-cells and to increase functional restoration of the neonatal brain after HI. Future research is needed to assess the influence of HT, sex and adjuvant therapies to cooling on neonatal brain injury so that precise and better neuroprotective and/or neuroregenerative therapies can be developed.

### Supplementary Information

Supplementary fig

## Data Availability

The datasets generated during and/or analysed during the current study are available from the corresponding author on reasonable request.
