# Supplementary figures and images for: Therapeutic hypothermia modulates the neurogenic response of the newborn piglet subventricular zone after hypoxia-ischemia
Source: Pediatr Res. 2023 Aug 12;95(1):112–9. doi: 10.1038/s41390-023-02751-7 (PMC10798892; doi:10.1038/s41390-023-02751-7)

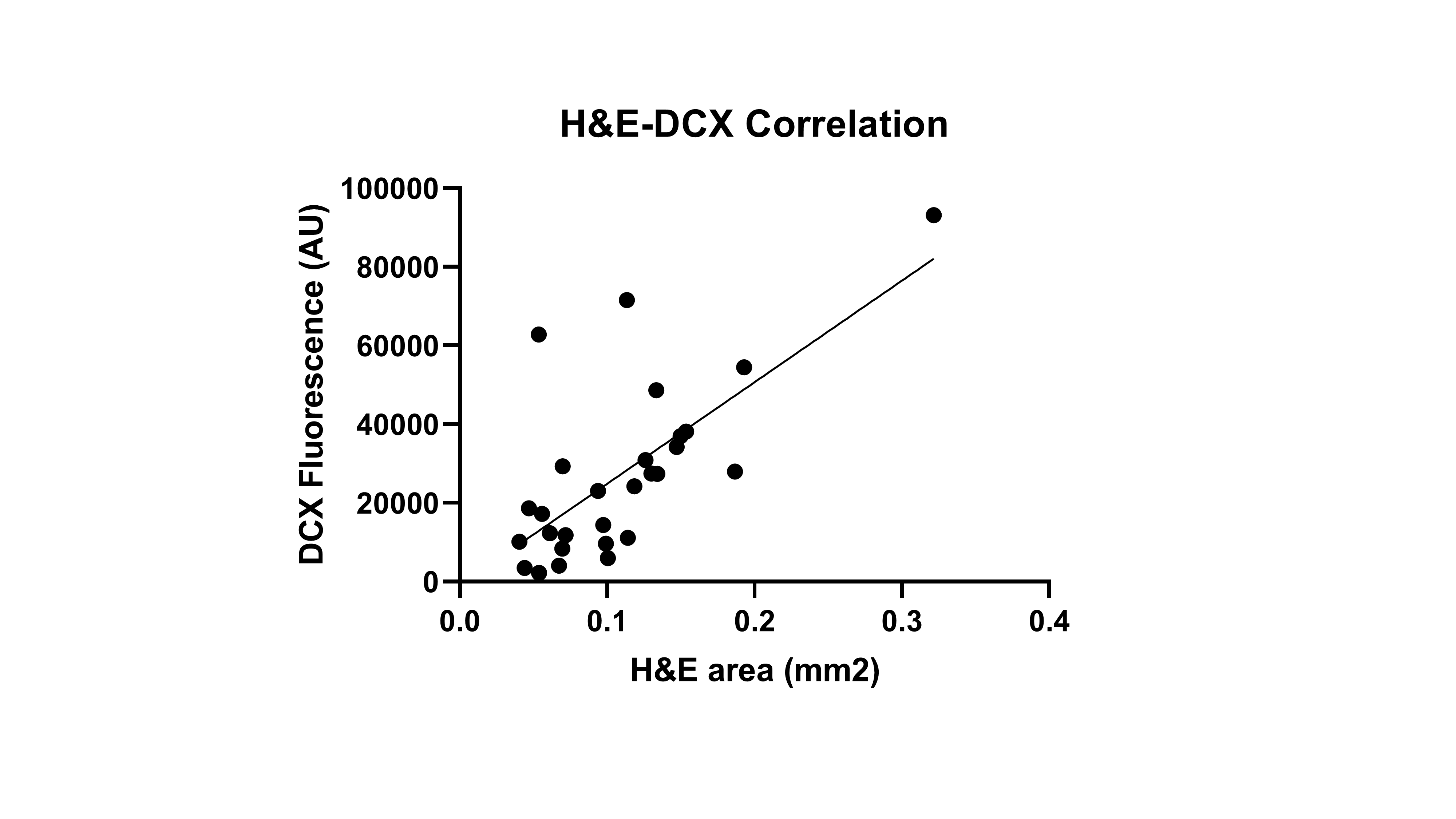

Supplement: Supplementary file 1 — Supplementary fig [file 41390_2023_2751_MOESM1_ESM.tif]
